# Minimally invasive *versus* traditional inverted “L” approach for posterior cruciate ligament avulsion fractures: a retrospective study

**DOI:** 10.7717/peerj.13732

**Published:** 2022-07-14

**Authors:** Yao Zhao, Huihui Guo, Liang Gao, Chang Liu, Xinzhong Xu, Wendan Cheng

**Affiliations:** 1 The Second Affiliated Hospital of Anhui Medical University, Hefei, China, China; 2 Center for Clinical Medicine, Huatuo Institute of Medical Innovation (HTIMI), Berlin, Germany; 3 Anhui Provincial Armed Police General Hospital, Hefei, China

**Keywords:** Minimally invasive, Posterior cruciate ligament, Avulsion fracture, Traditional approach, Clinical effects

## Abstract

**Purpose:**

To evaluate the clinical efficacy of a minimally invasive arthroscopic approach and to compare it with the traditional inverted “L” approach for the treatment of posterior cruciate ligament (PCL) avulsion fractures.

**Methods:**

From January 2016 to January 2020, the clinical data from patients with PCL avulsion fracture of the tibial insertion were analyzed retrospectively. They were divided into two groups based on surgical approaches: minimally invasive approach group (*n* = 15) and traditional inverted “L” group (*n* = 15 cases). The operation time, incision length, intraoperative blood loss, hospitalization time and complications were all recorded and compared between the two groups. The fracture healing time, knee range of motion (ROM), and residual relaxation degree were compared between the two groups after regular follow-up. The International Knee Documentation Committee (IKDC) and Lysholm scores were used to assess knee joint function.

**Results:**

There were no significant differences between the two groups in terms of gender, age, side, body mass index, cause of injury, Meyers McKeever classification and time from injury to operation (*P* > 0.05). The incision length and intraoperative bleeding in the minimally invasive group were significantly lower (*P* < 0.05) than those in the traditional group. There were no significant differences between the two groups in terms of operative time, fracture healing time, or residual relaxation (*P* > 0.05). The Lachman test and posterior drawer test were both negative, and there were no postoperative complications. The VAS pain score within 2 weeks and ROM within 4 weeks in the minimally invasive group were significantly better (*P* < 0.05) than those in the traditional inverted “L” approach group. The knee joint stability of both groups was good 12 months after surgery, and there were no significant differences in IKDC score, Lysholm score and ROM (*P* > 0.05) between the two groups.

**Conclusion:**

The minimally invasive approaches for the treatment of PCL avulsion fractures provide adequate exposure without the surgical complications associated with traditional open surgical approaches. The procedure is safe, fast and minimally invasive, and does not need a long learning curve.

## Introduction

As the most complex joint in the human body, knee stability depends to a great extent on its surrounding ligaments (Mehl et al., 2019). The posterior cruciate ligament (PCL) is an essential ligament for maintaining joint stability, and it originates from the anterior and upper part of the lateral wall of the medial femoral condyle and inserts at the posterior slope of the central tibial intercondylar area. The PCL connects the femur and tibia in a hinged manner. A biomechanical study revealed that the main function of the PCL is limited backwards relative to the femur. Tibia PCL avulsion fractures can improve the femur tibia backwards, which may greatly affect the stability of the knee joint after an injury (Bernhardson et al., 2019; Kew & Miller, 2020).

PCL injuries have been reported to account for 3–44% of all acute knee injuries, and are often associated with other ligament injuries (Fanelli & Edson, 1995; Shelbourne, Davis & Patel, 1999). The reported incidence has increased in recent years (Mariani et al., 2003). PCL avulsion fracture is a rare form of PCL injury compared to a typical PCL tear (Sonin et al., 1994), which is usually caused by high-energy mechanisms. The PCL is under the greatest tension during flexion and is prone to avulsion injury if subjected to forces on the anterior and posterior proximal tibia, such as an impact with the knee in flexion or a traffic accident (Bali et al., 2012). There are three types of avulsion fractures according to the Meyers–McKeever classification: Type I: undisplaced or slightly displaced anterior edge; Type II: the anterior part of the fracture is displaced and the posterior hinge side is intact; and Type III: complete displacement. If the avulsed fragment is not displaced, non-operative treatment may be recommended. Type II and III fractures usually require surgical reduction and internal fixation. Conservative treatment of an avulsion fracture with displacement of the tibial attachment often leads to fracture nonunion, painful walking with instability, and difficulty in bending (Keyhani, Soleymanha & Salari, 2020; Torisu, 1977). Therefore, surgical reduction and internal fixation are often required (Forkel et al., 2020). Surgical treatment can be accomplished by open reduction and internal fixation at the back of the knee (or posterior aspect of the knee) either with a traditional “L”-shaped incision (Burks & Schaffer, 1990) or with a minimally invasive arthroscopic approach (Zheng et al., 2021; Zhao et al., 2021a). Currently, there are a variety of treatment approaches for PCL avulsion fractures, but none of them has yet emerged as a gold-standard surgical technique (Malempati et al., 2015). This study aimed to compare the clinical efficacy of the minimally invasive posterior knee surgical approach and traditional inverted “L” approach for the treatment of PCL avulsion fractures.

## Methods

### Clinical data

#### Patient selection criteria

The inclusion criteria were as follows: (1) fresh PCL tibial avulsion fracture; (2) Meyers–McKeever class II and III fracture; (3) preoperative CT measurement of bony dimensions were used to fix the bony dimensions with cannulated lag screws; (3) good knee function before injury; (4) patient follow-up for more than 12 months, with complete imaging data; and (5) the imaging showed an average PCL fracture displacement of ≥6.7 mm (Yoon, Kim & Park, 2021). The degree of displacement of the fracture fragment was measured by KT-2000. The exclusion criteria were as follows: (1) patients with anterior cruciate ligament and collateral ligament injury; (2) the time from injury to operation was >2 weeks; (3) patients with preoperative joint dysfunction or history of knee trauma; and (4) patients with distal femoral or proximal tibial fractures.

### General information

In this retrospective study, 30 patients who met the screening criteria were recruited from Anhui Medical University between January 2016 and January 2020. All patients were enrolled by random number method, and written consent was obtained from all patients. According to different internal fixation methods, all patients were randomly divided into a minimally invasive approach group (*n* = 15) and traditional inverted “L” approach group (*n* = 15). There were no significant intergroup differences in demographic data such as age, sex, affected side, body mass index, cause of injury, residual fracture relaxation before surgery, Meyers–McKeever classification, and time from injury to operation (*P* > 0.05; Table 1).

**Table 1:** Comparison of general data between two groups.

Group	*N*	Gender		Side		Meyers-McKeever typing		Cause of injury		
		Male	Female	Left	Right	II	III	Treffic accident	Fall	Sports injury
Minimally invasive	15	6	9	8	7	4	11	10	3	2
Traditional inverted “L”	15	8	7	5	10	6	9	9	4	2
Statistc										
*P*-value		0.715		0.462		0.700		1.000		

Group	Age	BMI (kg/m2)	Time from injury to operation (days)	Preop IKDC score	Preop Lysholm score	Preop residual laxity
Minimally invasive	34.9 ± 8.1	22.1 ± 1.5	3.5 ± 1.0	45.1 ± 5.2	43.1 ± 2.9	14.5 ± 3.6
Traditional inverted “L”	35.1 ± 7.5	22.1 ± 1.8	3.7 ± 1.2	45.4 ± 4.8	43.4 ± 3.1	14.8 ± 3.6
Statistc	t = −0.073	t = −0.163	t = −0.286	t = −0.226	t = −0.219	−0.168
*P*-value	0.943	0.872	0.779	0.825	0.830	0.869

### Ethical approval

This clinical study was reviewed and approved by the Ethics Committee of the Second Affiliated Hospital of Anhui Medical University (approval of the prospective registry used in this study, project number: YX2020-092 [F1]).

### Surgical procedure

The patient was in the prone position, under general anesthesia, and with a pneumatic tourniquet applied proximally at the thigh. The tourniquet pressure was set at 280 mmHg, and the knee joint was flexed at 30°–45° to fully relax the medial gastrocnemius muscle. An inverted “L” incision (3–4 or 5–7 cm) was marked along the medial edge of the gastrocnemius muscle at 1–2 cm on the transverse striae of the popliteal fossa, followed by routine disinfection and towel spreading. Details of the two methods are provided below:

***With the traditional approach***, an “L”-shaped incision (5–7-cm long) was made at the postero-medial aspect of the knee. Its horizontal end was close to the medial flexion crease of the knee with the opposite end near the medial head of the gastrocnemius muscle (Burks & Schaffer, 1990).

After separating the subcutaneous tissue and reaching the deep fascia, the medial head of the gastrocnemius was identified, and an interval was formed between the medial gastrocnemius and the semitendinosus muscles. After blunt dissection into this interval with the knee flexed at 30°, the medial head of the gastrocnemius, popliteal vessels, and nerve were moved laterally to the outer side to prevent traction on the neurovascular structures, thus fully exposing the posterior knee capsule.

***With the minimally invasive approach***, a horizontal skin incision (3–4 cm) began at the medial side of the popliteal artery towards the lateral side. The deep fascia was also incised to expose the medial head of the gastrocnemius muscle. With the knee in 30° flexion, the space between the gastrocnemius and semitendinosus was dissected, and the medial head of the gastrocnemius pulled laterally using a thyroid retractor until reaching the posterior capsule. By exposing the capsule, the thick muscular belly that protects the vascular and neural structures was moved aside without directly exerting tension on the neural structures. The capsule was then incised longitudinally to uncover the avulsed bony fragment, which was lifted along with the PCL to maintain its continuity. After thoroughly washing the operation site with normal saline, the fractured block was reset to the bone bed, and one or two Kirschner wires were placed in the direction perpendicular to the fractured block according to the size of the fractured block. Under fluoroscopy, the direction and depth of the Kirschner wires were adjusted. Two partially threaded lag screws (4.5 mm) were used depending on the bone fragment size. After assuring proper fixation, the joint was thoroughly irrigated and the capsule was sutured. A suction drain tube was positioned and the wound was closed in layers. The knee was then placed on an adjustable brace at 30° flexion to minimize tension forces over the PCL.

### Postoperative rehabilitation

Ankle pump exercise, quadriceps contraction, and other functional exercises were started on the day after surgery. Further, straight leg raises were also begun the day after surgery. Toe contact or partial weight-bearing was allowed for the first 4 weeks after surgery. Passive range of motion (ROM, 0°–60°) was achieved over the next 3 weeks, active flexion in the prone position (0°–90°) was achieved between 4 and 6 weeks, and complete ROM was attempted after 8 weeks. Twelve weeks postoperatively, the patients walked with a brace to strengthen the quadriceps muscles and improve mobility. Returning to activities of daily living occurred within 6 months postoperatively, while returning to physical work and sports gradually resumed within 8–12 months. Radiographs of the knees were taken every month after surgery to determine whether the fracture healing was in line with clinical criteria.

The reduction/union of the fracture, as well as the stability and ROM of the knee joint, were all monitored on a regular basis. The Lysholm knee function scoring standard was used to assess the overall functional recovery of the affected limbs.

### Statistical analysis

SPSS 23.0 (IBM Corporation, Armonk, NY, USA) statistics software was used for statistical analysis. Normally distributed measurement data were expressed as mean ± standard deviation, and independent-sample *t*-test was used for comparison between groups. Non-normally distributed measurement data were presented as median (interquartile range), and rank-sum test was used for comparison between groups. Statistical data were expressed as rates, and chi-squared test or Fisher’s exact probability method was used for comparison between groups. Rank sum test was used for comparison of grade data between groups. A *P* value of <0.05 was considered statistically significant.

## Results

### Comparison of general postoperative characteristics between the two groups

Both groups had successful operations, and the incisions healed completely. No postoperative complications such as incision infection, intraarticular infection, joint fibrosis, deep vein thrombosis, or fracture nonunion occurred in either group. The postoperative Lachman test and back drawer test were negative, and the knee joint stability showed good recovery. There were no significant differences between the two groups in terms of operation time, postoperative residual fracture relaxation, postoperative hospital stay, fracture healing time, and follow-up time (*P* > 0.05; Table 2). Incision length and intraoperative blood loss were significantly lower in the minimally invasive approach group than in the traditional “inverted L” approach group (*P* < 0.05; Table 2).

**Table 2:** Comparison of clinical evalution indexes between two groups after operation.

Group	*N*	Operation time (min)	Incision length (cm)	Intraoperative blood loss (ml)	Hospitalization stay (day)	Follow-up time (months)
Minimally invasive	15	43.2 ± 5.3	3.7 ± 0.7	49.0 ± 5.7	3.5 ± 0.7	14.9 ± 1.7
Traditional inverted “L”	15	45.4 ± 5.5	6.3 ± 0.9	80.2 ± 10.9	3.7 ± 0.8	14.5 ± 1.6
Statistc		t = −0.978	t = −10.217	−9.723	t = −1.000	t = −0.823
*P*-value		0.345	0.000	0.000	0.334	0.424

Group	*N*	Range of motion (°)	Lysholm score	IKDC score	Residual laxity	Healing time (months)
Minimally invasive	15	135.1 ± 4.7	92.8 ± 3.2	93.9 ± 2.4	2.1 ± 0.8	3.0 ± 0.2
Traditional inverted “L”	15	134.1 ± 4.0	92.5 ± 3.5	93.5 ± 3.1	1.9 ± 0.8	3.2 ± 0.2
Statistc		0.951	0.195	0.423	−0.487	−1.773
*P*-value		0.358	0.848	0.679	0.634	0.098

**Figure 1:**
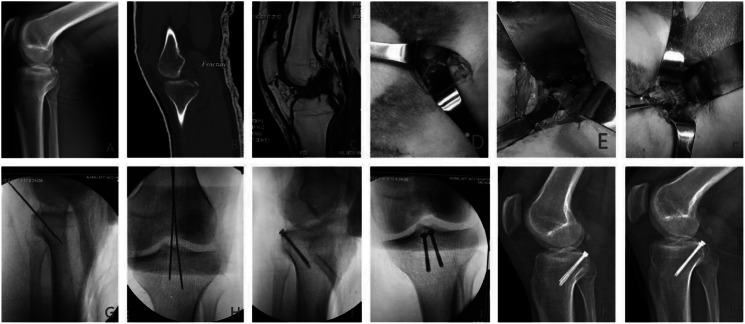
Preoperative, intraoperative and postoperative images. (A–C) Preoperative radiograph, CT, and MRI; (D–F) the gastrocnemius muscle was separated and pulled laterally to the joint capsule, the joint capsule was opened to observe the fracture mass, and Kirschner wire fixation was performed intraoperatively (as indicated by the arrow); (G–J) anteroposterior-lateral radiograph after intraoperative Kirschner wire fixation and screw placement showed good fracture reduction and internal fixation position; (K–l) lateral radiographs at 3 and 12 months postoperatively showed union.

**Figure 2:**
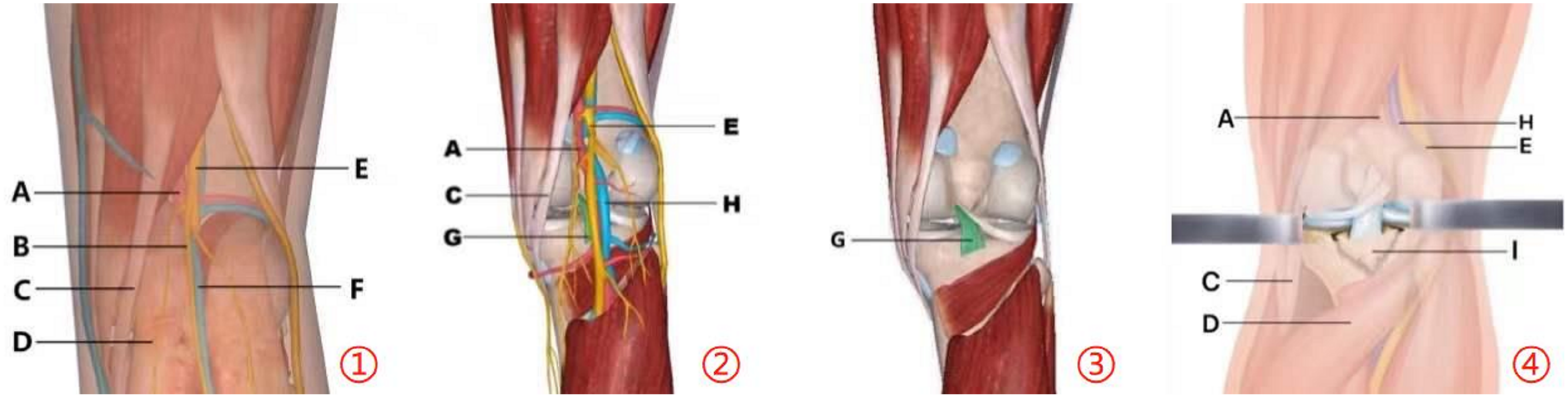
Surgical anatomical illustration of PCL avulsion fracture of tibial insertion. ①–③ show the anatomical structures from superficial to deep layers within the surgical area; ④ shows the approach to expose the fracture site with the medial head of the gastrocnemius pulled laterally. (A) Popliteal artery; (B) medial gastrocnemius cutaneous nerve (C) semitendinosus; (D) gastrocnemius; (E) tibial nerve; (F) small saphenous vein; (G) posterior cruciate ligament; (H) popliteal vein; (I) avulsion fracture of posterior cruciate ligament.

Preoperative, intraoperative, and postoperative imaging data are shown in Fig. 1. The schematic diagram of the operation is shown in Fig. 2.

### The IKDC score, Lysholm score, and ROM of knee fracture block were compared 12 months after surgery between the two groups

Both groups achieved good results 12 months after surgery, indicating good fracture healing and normal knee function, according to the 12-month follow-up. There was no statistically significant difference between the two groups (*P* > 0.05; Table 2). The findings demonstrated that patients’ fractures could be successfully treated using two surgical approaches.

### Visual analog scale (VAS) pain scores were compared between the two groups at various postoperative time points

At 1, 3, 7, and 14 days after surgery, the VAS pain score of the minimally invasive approach group was lower than that of the traditional inverted “L” group (*P* < 0.05, Table 3). Patients in the minimally invasive approach group likely have smaller surgical incisions, less dissection of damaged peripheral blood vessels and nerves during surgery, and less damage to the surrounding soft tissues than patients in the traditional inverted “L” approach group, resulting in less overall postoperative pain.

**Table 3:** Comparison of VAS pain scores in patients with avulsion fracture of PCL tibial at different postoperative time points ( ${\bar X}$ ± S,points).

Group	*N*	1 day post-op	3 days post-op	1 week post-op	2 weeks post-op
Minimally invasive	15	6.9 ± 1.0	4.5 ± 0.9	2.5 ± 0.9	1.1 ± 0.9
Traditional inverted “L”	15	7.7 ± 0.8	5.1 ± 0.2	3.1 ± 0.9	1.7 ± 0.8
Statistc		t = −2.256	t = −2.358	t = −2.197	−2.201
*P*-value		0.041	0.033	0.045	0.045

### Comparison of knee motion in the two groups at various postoperative times

The knee motion of the minimally invasive approach group was significantly higher than that of the traditional inverted “L” group at 1, 2, and 4 weeks after surgery, with statistical significances (*P* < 0.05, Table 4). After a 12-month follow-up in which both groups of patients were compared with no statistically significant difference (*P* > 0.05, Table 4), due to the obvious small surgical incision, less damage to peripheral blood vessels and soft tissue during the operation, light pain for patients in the early postoperative period, and early functional exercise, the minimally invasive approach group outperformed the traditional inverted “L” approach group. Patients in both groups achieved the clinical healing standard over time, and knee joint activity returned to normal.

**Table 4:** Comparison of ROM at different postoperative time points in patients with avulsion fracture of PCL tibial ( ${\bar X}$ ± S,°).

Group	*N*	1 week post-op	2 weeks post-op	4 weeks post-op	8 weeks post-op
Minimally invasive	15	22.9 ± 2.6	44.6 ± 3.9	74.5 ± 2.9	131.4 ± 3.5
Traditional inverted “L”	15	20.7 ± 1.8	41.6 ± 2.9	71.2 ± 3.8	131.1 ± 3.5
Statistc		2.930	2.393	2.352	0.326
*P*-value		0.011	0.031	0.034	0.750

## Discussion

The most important finding of this study was that, based on the functional and radiographic evaluations, minimally invasive fixation approach for treatment of acute displaced PCL avulsion fractures could achieve comparably better clinical outcomes, radiologic healing, and knee stability at intermediate follow-up.

The PCL is critical in keeping the knee stable. Its primary function is to prevent excessive knee hyperextension and rotation. PCL avulsion fracture is a type of PCL injury that causes knee flexion and rotation instability; increases the lateral ROM, along with hyperextension and hyperflexion of the knee joint; and increases pressure on the patellofemoral surface (Hooper et al., 2018b; Huang et al., 2015). Long-term changes can impair knee joint function and accelerate meniscus wear, resulting in degenerative changes in the joint and a poor quality of life. Therefore, early treatment and rapid recovery are paramount to good prognosis and outcome (Zhu et al., 2017).

The conventional open approaches used for fixing PCL avulsions can be divided into three types based on the direction of access: midline, medial, and lateral. Burks and Schaffer’s inverted “L” type is one of the most commonly used traditional surgical treatments for PCL avulsion fractures of tibial insertion (Burks & Schaffer, 1990). This approach can expose the PCL attachment site, and it is not necessary to separate the popliteal blood vessels and nerves during the operation, but the medial gastrocnemius tendon still needs to be severed. The exposure to the lateral base of the PCL is limited, and screw placement perpendicular to the fracture plane is difficult, thereby affecting the fixation stability. Furthermore, Trickey (1968) described and modified the “S”-shaped incision, which is located in the posterior midline of the knee. It is fully exposed to the posterior area of the knee, but is longer and has the potential to damage the popliteal fossa vascular nerves, thus increasing the risk of postoperative bleeding. Gavaskar et al. (2017) proposed the lateral popliteal incision, which depends on the accuracy of the skin incision. A wrong incision can make the operation difficult. The midline incision is at risk of nerve and blood vessel injury. Keyhani, Soleymanha & Salari (2020) adopts the direct posterior approach, which does not need to separate the medial head of the gastrocnemius muscle to improve the exposure of PCL avulsion. However, the incision made by this approach is large, and improper manipulation of the popliteal midline can easily damage blood vessels and nerves.

The current technique employs the same plane described previously by several authors between the two heads of the gastrocnemius muscle. It has been demonstrated that the smaller incision, when placed correctly, provides adequate exposure of the posterior capsule for at least 2.5 cm on either side of the midline to perform a satisfactory fracture fixation.

The minimally invasive approach has the following advantages over the traditional inverted “L” approach. (1) The surgical incision is 3–4 cm long, which is shorter than that in the traditional approach (7 cm). It not only meets the patient’s aesthetic needs but also helps to reduce intraoperative bleeding (about 50 mL). (2) Postoperative pain is reduced, but it is also conducive to early functional exercise, which promotes early recovery. (3) The surgical approach uses natural anatomical spaces. Hence, the tissue is easy to separate and blood vessel ligation is not required, thereby saving surgical time and lowering the risk of iatrogenic injury when compared to the traditional approach. (4) The surgery has a shorter learning curve than the arthroscopic technique, making it more suitable for surgeons working in hospitals without specialized equipment (Zhao et al., 2021b).

With the progress of arthroscopic technology in recent years, an increasing number of scholars at China and abroad have begun to use arthroscopy for the treatment of PCL avulsion fractures (Sabat, Jain & Kumar, 2016; Yoon, Park & Lee, 2018). The arthroscopic approach is less invasive and safer, allowing for early functional exercise; moreover, for patients with combined meniscus injury, it can be performed at the same time, making it a more patient-friendly approach. However, anatomical reduction is difficult to achieve by arthroscopic surgery, and the learning curve and cycle are relatively long (Hooper et al., 2018b). Furthermore, due to the need for specialized surgical equipment, operations cannot be performed in primary care hospitals (Hooper et al., 2018a; Rhee et al., 2019). Compared with arthroscopy, the minimally invasive technique described herein reduces the incidence of traditional open surgery, and does not require a long learning curve.

Nevertheless, the small sample size of this study limits its ability to prove whether minimally invasive surgery is appropriate for all PCL avulsion fractures. Therefore, the findings of our study can only provide a portion of the clinical treatment of minimally invasive approach. Different internal fixation materials and surgical methods should be used depending on the patient’s fracture block situation. Furthermore, X-ray fluoroscopy can be used to locate the surgical incision location prior to surgery in order to reduce surgical incision error and avoid prolonging the incision or switching to a traditional approach due to incision location problems during surgery. In this study, the BMI of patients undergoing surgery may have been lower than that of the western population, and the surgical incision required for morbidly obese patients may be larger. The operation time of minimally invasive arthroscopic approach is comparable to that of previous techniques, which we consider highly reproducible. Effective lag screw fixation can be accomplished without the extensive use of the technique, thus allowing for early mobilization.

## Conclusion

The minimally invasive technique provides adequate internal fixation for PCL avulsion fracture at the tibial insertion. This technology is simple to use, requires little time to set up, provides adequate exposure, and is safe. The postoperative pain relief is excellent, which is advantageous for early rehabilitation exercise program and knee joint function recovery. The method is reliable and safe and has promising potential for clinical applications.

## Supplemental Information

10.7717/peerj.13732/supp-1 Supplemental Information 1 Patient information.

## Additional Information and Declarations

### Data Availability

The following information was supplied regarding data availability: The original data is in the Supplemental figure and table.
