# Supplementary material for: Minimally invasive versus traditional inverted “L” approach for posterior cruciate ligament avulsion fractures: a retrospective study
Source: PeerJ. 2022 Jul 14;10:e13732. doi: 10.7717/peerj.13732 (PMC9288828; doi:10.7717/peerj.13732)
Supplement: Supplemental Information 1 [file peerj-10-13732-s001.docx]

Dataset

Characteristics and clinical data of patients with traditional inverted "L" method

| **Surgery time (min)** | **Healing time**  **(month)** | **Preop IKDC**  **score** | **Postop IKDC score** | **Preop**  **Lysholm**  **score** | **Postop Lysholm**  **score** | **Preop**  **ROM**  **(°)** | **Postop ROM**  **(°)** | **Preop Residual laxity**  **(mm)** | **Complications** |
| --- | --- | --- | --- | --- | --- | --- | --- | --- | --- |
| 43 | 3 | 38 | 88 | 44 | 91 | 65 | 132 | 17 | NO |
| 40 | 3.5 | 44 | 95 | 44 | 94 | 62 | 139 | 21 | NO |
| 39 | 3.3 | 46 | 96 | 40 | 88 | 56 | 129 | 16 | NO |
| 41 | 3 | 53 | 90 | 45 | 91 | 58 | 135 | 15 | NO |
| 42 | 2.8 | 41 | 92 | 43 | 95 | 57 | 128 | 18 | NO |
| 43 | 2.9 | 44 | 98 | 49 | 90 | 55 | 133 | 21 | NO |
| 55 | 3.4 | 43 | 95 | 42 | 95 | 63 | 128 | 9 | NO |
| 58 | 3.4 | 45 | 91 | 41 | 94 | 68 | 134 | 10 | NO |
| 48 | 3.3 | 52 | 90 | 38 | 87 | 63 | 140 | 11 | NO |
| 49 | 2.8 | 51 | 97 | 47 | 96 | 62 | 136 | 12 | NO |
| 47 | 2.9 | 50 | 95 | 39 | 94 | 61 | 133 | 14 | NO |
| 48 | 3.4 | 40 | 94 | 46 | 86 | 70 | 140 | 15 | NO |
| 42 | 3.4 | 39 | 90 | 44 | 95 | 63 | 134 | 13 | NO |
| 43 | 3.1 | 48 | 97 | 42 | 95 | 60 | 138 | 14 | NO |
| 43 | 3.1 | 47 | 94 | 47 | 97 | 53 | 133 | 16 | NO |

| **Age** | **Body mass index(kg/m2)** | **Time from injury to operation(days)** | **Incision length(cm)** | **Intraoperative blood loss(ml)** | **Postop Residual laxity**  **(mm)** | **Hospitalization stay (day)** | **Follow-up time(months)** |
| --- | --- | --- | --- | --- | --- | --- | --- |
| 32 | 22 | 5 | 6 | 80 | 2 | 3 | 15 |
| 35 | 23 | 3 | 7 | 85 | 3 | 4 | 15 |
| 33 | 24 | 4 | 6 | 85 | 1 | 3 | 14 |
| 27 | 19 | 3 | 7 | 70 | 3 | 4 | 12 |
| 36 | 20 | 6 | 5 | 65 | 1 | 5 | 17 |
| 34 | 23 | 3 | 7 | 78 | 3 | 3 | 18 |
| 28 | 22 | 2 | 6 | 50 | 2 | 5 | 13 |
| 33 | 23 | 4 | 8 | 80 | 2 | 4 | 14 |
| 37 | 23 | 3 | 6 | 85 | 1 | 3 | 13 |
| 39 | 19 | 5 | 6 | 90 | 2 | 4 | 15 |
| 50 | 24 | 3 | 7 | 85 | 2 | 3 | 14 |
| 25 | 25 | 2 | 5 | 90 | 3 | 3 | 13 |
| 38 | 23 | 4 | 7 | 85 | 3 | 5 | 16 |
| 51 | 22 | 5 | 6 | 85 | 1 | 4 | 14 |
| 28 | 20 | 3 | 5 | 90 | 2 | 3 | 15 |

VAS pain score of the traditional inverted "L" method.

| one day post-op | There days post-op | One week post-op | Twoweeks post-op |
| --- | --- | --- | --- |
| 8 | 5 | 3 | 2 |
| 7 | 4 | 2 | 1 |
| 8 | 5 | 3 | 2 |
| 9 | 7 | 4 | 1 |
| 8 | 4 | 2 | 3 |
| 7 | 6 | 4 | 2 |
| 8 | 5 | 3 | 1 |
| 8 | 3 | 4 | 2 |
| 7 | 4 | 3 | 1 |
| 9 | 5 | 2 | 2 |
| 7 | 5 | 3 | 2 |
| 8 | 7 | 5 | 3 |
| 6 | 6 | 4 | 0 |
| 8 | 4 | 2 | 2 |
| 8 | 6 | 3 | 1 |

The range of motion of knee joint in the traditional inverted "L" method

| One week post-op | Two weeks post-op | Four weeks post-op | Eight weeks post-op |
| --- | --- | --- | --- |
| 22 | 43 | 70 | 128 |
| 20 | 42 | 68 | 133 |
| 21 | 42 | 71 | 126 |
| 25 | 44 | 69 | 133 |
| 19 | 41 | 67 | 125 |
| 20 | 38 | 72 | 130 |
| 21 | 47 | 73 | 127 |
| 20 | 39 | 68 | 133 |
| 19 | 36 | 76 | 135 |
| 20 | 41 | 77 | 134 |
| 21 | 46 | 69 | 130 |
| 23 | 40 | 68 | 136 |
| 18 | 43 | 71 | 129 |
| 22 | 40 | 80 | 135 |
| 19 | 42 | 69 | 133 |

Characteristics and clinical data of patients with minimally invasive approach

| **Surgery time (min)** | **Healing time**  **(month)** | **Preop IKDC**  **score** | **Postop IKDC score** | **Preop**  **Lysholm**  **score** | **Postop Lysholm**  **score** | **Preop**  **ROM**  **(°)** | **Postop ROM**  **(°)** | **Postop Residual laxity**  **(mm)** | **Complications** |
| --- | --- | --- | --- | --- | --- | --- | --- | --- | --- |
| 44 | 2.8 | 41 | 93 | 44 | 89 | 64 | 137 | 18 | NO |
| 58 | 3 | 39 | 91 | 43 | 93 | 54 | 136 | 12 | NO |
| 42 | 2.9 | 40 | 94 | 37 | 94 | 55 | 128 | 11 | NO |
| 38 | 3.3 | 37 | 92 | 44 | 95 | 62 | 132 | 10 | NO |
| 40 | 3.1 | 48 | 95 | 42 | 86 | 59 | 130 | 11 | NO |
| 42 | 3.2 | 49 | 96 | 41 | 95 | 58 | 135 | 8 | NO |
| 40 | 2.7 | 46 | 89 | 43 | 92 | 63 | 132 | 15 | NO |
| 42 | 2.9 | 47 | 95 | 48 | 96 | 62 | 138 | 16 | NO |
| 39 | 2.8 | 53 | 96 | 46 | 93 | 64 | 140 | 14 | NO |
| 44 | 2.7 | 54 | 92 | 45 | 87 | 61 | 134 | 16 | NO |
| 42 | 3 | 50 | 98 | 47 | 94 | 56 | 140 | 18 | NO |
| 41 | 3.3 | 43 | 95 | 41 | 95 | 73 | 146 | 13 | NO |
| 53 | 3.4 | 40 | 97 | 39 | 91 | 63 | 133 | 17 | NO |
| 41 | 3 | 43 | 92 | 42 | 96 | 61 | 130 | 20 | NO |
| 42 | 3 | 46 | 94 | 45 | 96 | 64 | 136 | 19 | NO |

| **Age** | **Body mass index(kg/m2)** | **Time from injury to operation(days)** | **Incision length(cm)** | **Intraoperative blood loss(ml)** | **Postop Residual laxity**  **(mm)** | **Hospitalization stay (day)** | **Follow-up time(months)** |
| --- | --- | --- | --- | --- | --- | --- | --- |
| 38 | 24 | 4 | 3 | 45 | 3 | 3 | 12 |
| 26 | 23 | 5 | 3 | 50 | 1 | 4 | 16 |
| 30 | 24 | 3 | 4 | 40 | 1 | 3 | 14 |
| 37 | 20 | 4 | 4 | 55 | 2 | 3 | 15 |
| 29 | 22 | 2 | 3 | 50 | 1 | 3 | 13 |
| 37 | 22 | 5 | 4 | 45 | 3 | 3 | 18 |
| 43 | 21 | 3 | 4 | 50 | 2 | 5 | 14 |
| 40 | 23 | 4 | 5 | 40 | 3 | 4 | 15 |
| 33 | 22 | 3 | 4 | 55 | 2 | 5 | 16 |
| 52 | 21 | 2 | 3 | 60 | 2 | 3 | 15 |
| 34 | 23 | 5 | 3 | 45 | 3 | 4 | 14 |
| 25 | 24 | 3 | 5 | 45 | 2 | 3 | 13 |
| 46 | 19 | 4 | 4 | 55 | 1 | 3 | 17 |
| 29 | 22 | 3 | 3 | 50 | 2 | 3 | 15 |
| 24 | 21 | 3 | 3 | 50 | 1 | 3 | 17 |

VAS pain score of the minimally invasive approach.

| one day post-op | There days post-op | One week post-op | Twoweeks post-op |
| --- | --- | --- | --- |
| 7 | 4 | 4 | 2 |
| 6 | 5 | 3 | 1 |
| 8 | 4 | 2 | 3 |
| 7 | 4 | 4 | 1 |
| 8 | 3 | 1 | 0 |
| 6 | 6 | 2 | 1 |
| 6 | 5 | 1 | 0 |
| 7 | 4 | 2 | 1 |
| 9 | 4 | 3 | 1 |
| 7 | 5 | 2 | 2 |
| 8 | 4 | 3 | 0 |
| 5 | 6 | 2 | 1 |
| 7 | 5 | 3 | 0 |
| 6 | 3 | 2 | 2 |
| 7 | 5 | 3 | 1 |

The range of motion of knee joint in the traditional inverted "L" method

| One week post-op | Two weeks post-op | Four weeks post-op | Eight weeks post-op |
| --- | --- | --- | --- |
| 20 | 45 | 72 | 130 |
| 25 | 47 | 75 | 131 |
| 24 | 48 | 80 | 125 |
| 26 | 43 | 73 | 128 |
| 27 | 50 | 77 | 129 |
| 23 | 38 | 78 | 134 |
| 19 | 38 | 73 | 130 |
| 21 | 42 | 76 | 135 |
| 23 | 46 | 74 | 136 |
| 25 | 39 | 73 | 130 |
| 24 | 49 | 78 | 134 |
| 21 | 48 | 69 | 138 |
| 18 | 44 | 75 | 128 |
| 24 | 45 | 71 | 130 |
| 23 | 47 | 73 | 133 |
